# Supplementary material for: Deep Learning-Based Muscle Segmentation and Quantification of Full-Leg Plain Radiograph for Sarcopenia Screening in Patients Undergoing Total Knee Arthroplasty
Source: J Clin Med. 2022 Jun 22;11(13):3612. doi: 10.3390/jcm11133612 (PMC9267147; doi:10.3390/jcm11133612)
Supplement: Supplementary file 1 [file jcm-11-03612-s001.zip › jcm-1781001-supplementary.pdf]

Table S1. Baseline characteristics and sarcopenia markers of sarcopenic and non-sarcopenic patients.

Characteristics	All patients (N=403)	Sarcopenic (N=34)	Normal (N=369)	*p*-value
Demographics				
Age at surgery, year (SD)	70.88 (6.59)	74.62 (6.52)	70.53 (6.50)	<0.001
Sex (%)				
Female	351 (87.1)	32 (94.1)	319 (86.4)	0.266
Male	52 (12.9)	2 (5.9)	50 (13.6)	
Height, cm (SD)	154.36 (6.58)	149.41 (5.88)	154.82 (6.46)	<0.001
Weight, kg (SD)	63.19 (9.30)	53.44 (8.43)	64.08 (8.86)	<0.001
BMI, kg/m^2 (SD)	26.50 (3.26)	23.86 (3.36)	26.72 (3.16)	<0.001
Sarcopenia Markers				
Predicted muscle volume, cm^3 (SD)	8296.4 (1660.9)	6972.4 (1354.6)	8418.4 (1634.8)	<0.001
Comorbidities				
ASA class				0.197
0	7 (1.7)	1 (2.9)	6 (1.6)	
1	68 (16.9)	7 (20.6)	61 (16.5)	
2	304 (75.4)	22 (64.7)	282 (76.4)	
3	24 (6.0)	4 (11.8)	20 (5.4)	
Hypertension (%)	154 (38.2)	8 (23.5)	146 (39.6)	0.068
Diabetes mellitus (%)	81 (20.1)	11 (32.4)	70 (19.0)	0.074

Values are shown as the mean ± standard deviation or number (%). Statistical significance was set at $p<0.05$.

ASA, American society of anesthesia

Table S2. Laboratory data of sarcopenic and non-sarcopenic patients.

Characteristics	All patients (N=403)	Sarcopenic (N=34)	Normal (N=369)	*p*-value
PT INR (SD)	0.96 (0.06)	0.95 (0.04)	0.96 (0.07)	0.228
Hemoglobin, g/dL (SD)	13.00 (1.74)	12.25 (1.15)	13.07 (1.77)	0.008
Platelet count × /10^9L (SD)	239.64 (57.86)	251.62 (51.00)	238.54 (58.39)	0.208
ESR, mm/h (SD)	18.56 (12.98)	15.71 (10.66)	18.82 (13.15)	0.180
C-Reactive Protein, mg/dL (SD)	0.19 (0.47)	0.10 (0.11)	0.20 (0.49)	0.248
Albumin, g/dL (SD)	4.22 (0.35)	4.14 (0.25)	4.23 (0.36)	0.194
Total Protein, mg/dL (SD)	7.02 (0.44)	6.73 (0.42)	7.05 (0.43)	<0.001
AST(GOT), mg/dL (SD)	24.37 (13.86)	21.85 (4.43)	24.60 (14.41)	0.269
ALT(GPT), mg/dL (SD)	21.76 (11.28)	18.71 (8.10)	22.05 (11.50)	0.099
Alkaline phosphatase, mg/dL (SD)	71.57 (22.91)	67.15 (21.46)	71.98 (23.03)	0.240
Total Bilirubin, mg/dL (SD)	0.62 (0.24)	0.61 (0.25)	0.62 (0.24)	0.946
BUN, mg/dL (SD)	18.39 (6.01)	20.32 (10.24)	18.22 (5.45)	0.050
Creatinine, mg/dL (SD)	0.79 (0.24)	0.79 (0.44)	0.79 (0.21)	0.953
eGFR – CKD EPI (SD)	80.13 (14.38)	79.62 (17.40)	80.18 (14.09)	0.828
Sodium, mg/dL (SD)	140.86 (7.30)	141.59 (1.84)	140.79 (7.60)	0.542
Potassium, mg/dL (SD)	4.31 (0.43)	4.36 (0.41)	4.31 (0.43)	0.498
Chloride, mg/dL (SD)	105.01 (2.40)	105.06 (2.41)	105.00 (2.40)	0.896
Total Calcium, mg/dL (SD)	9.22 (0.42)	9.27 (0.45)	9.21 (0.42)	0.431
Phosphorus, mg/dL (SD)	3.67 (0.50)	3.68 (0.48)	3.67 (0.50)	0.934
Uric acid, mg/dL (SD)	4.79 (1.21)	4.39 (1.27)	4.83 (1.20)	0.040
Glucose, mg/dL (SD)	123.56 (37.79)	129.18 (42.62)	123.05 (37.34)	0.366

Values are shown as the mean ± standard deviation (SD). Statistical significance was set at $p<0.05$.

ESR, Erythrocyte Sedimentation Rate; BUN, Blood Urea Nitrogen

Table S3. Performance assessment of the model on Cohort A (training set) and Cohort B (test set).

Population	DSC	Mean IoU
Cohort A	0.944 [0.936, 0.951]	0.959 [0.959, 0.960]
1 - fold	0.942	0.959
2 - fold	0.937	0.959
3 - fold	0.940	0.959
4 - fold	0.938	0.960
5 - fold	0.963	0.960
Cohort B	0.913 [0.910, 0.916]	0.926 [0.920, 0.931]

The brackets indicate the 95% confidence interval. Dice Similarity Coefficient; IoU, Intersection-over-Union.
